# Evidence for Stress-like Alterations in the HPA-Axis in Women Taking Oral Contraceptives

**DOI:** 10.1038/s41598-017-13927-7

**Published:** 2017-10-26

**Authors:** Johannes Hertel, Johanna König, Georg Homuth, Sandra Van der Auwera, Katharina Wittfeld, Maik Pietzner, Tim Kacprowski, Liliane Pfeiffer, Anja Kretschmer, Melanie Waldenberger, Gabi Kastenmüller, Anna Artati, Karsten Suhre, Jerzy Adamski, Sönke Langner, Uwe Völker, Henry Völzke, Matthias Nauck, Nele Friedrich, Hans Joergen Grabe

**Affiliations:** 1 Department of Psychiatry and Psychotherapy, University Medicine Greifswald, Greifswald, Germany; 2 German Center for Neurodegenerative Diseases (DZNE), Site Rostock/Greifswald, Greifswald, Germany; 3 Institute of Clinical Chemistry and Laboratory Medicine, University Medicine Greifswald, Greifswald, Germany; 4 DZHK (German Center for Cardiovascular Research), partner site Greifswald, Greifswald, Germany; 5 Institute for Community Medicine, University Medicine Greifswald, Greifswald, Germany; 6 Interfaculty Institute for Genetics and Functional Genomics, University Medicine Greifswald, Greifswald, Germany; 7 Research Centre for Prevention and Health, Glostrup University Hospital, Glostrup, Denmark; 8 Research Unit of Molecular Epidemiology, Helmholtz Zentrum München, German Research Center for Environmental Health, Neuherberg, Germany; 9 Institute of Epidemiology II, Helmholtz Zentrum München, German Research Center for Environmental Health, Neuherberg, Germany; 10 Institute of Bioinformatics and Systems Biology, Helmholtz Zentrum München, Neuherberg, Germany; 11 IEG (Institute of Experimental Genetics), Genome Analysis Center, Helmholtz Zentrum München, Neuherberg, Germany; 12 Weill Cornell Medical College in Qatar, Education City, Qatar Foundation, Doha, Qatar; 13 DZD (German Center for Diabetes Research), site München-Neuherberg, Neuherberg, Germany; 14 Lehrstuhl für Experimentelle Genetik, Technische Universität München, Freising-Weihenstephan, Germany; 15 Institute of Diagnostic Radiology, University Medicine Greifswald, Greifswald, Germany

## Abstract

Using oral contraceptives has been implicated in the aetiology of stress-related disorders like depression. Here, we followed the hypothesis that oral contraceptives deregulate the HPA-axis by elevating circulating cortisol levels. We report for a sample of 233 pre-menopausal women increased circulating cortisol levels in those using oral contraceptives. For women taking oral contraceptives, we observed alterations in circulating phospholipid levels and elevated triglycerides and found evidence for increased glucocorticoid signalling as the transcript levels of the glucocorticoid-regulated genes *DDIT4* and *FKBP5* were increased in whole blood. The effects were statistically mediated by cortisol. The associations of oral contraceptives with higher *FKBP5* mRNA and altered phospholipid levels were modified by rs1360780, a genetic variance implicated in psychiatric diseases. Accordingly, the methylation pattern of *FKBP5* intron 7 was altered in women taking oral contraceptives depending on the rs1360780 genotype. Moreover, oral contraceptives modified the association of circulating cortisol with depressive symptoms, potentially explaining conflicting results in the literature. Finally, women taking oral contraceptives displayed smaller hippocampal volumes than non-using women. In conclusion, the integrative analyses of different types of physiological data provided converging evidence indicating that oral contraceptives may cause effects analogous to chronic psychological stressors regarding the regulation of the HPA axis.

## Introduction

Oral contraceptive (OC) medication belongs to the World Health Organization’s List of Essential Medicines^1^ and thus is supposed to be integral to a modern health system. In 2015, according to the United Nations (UN), approximately 70 million women worldwide prevented conception using OCs with OCs clearly being the method of choice for Western Europe^2^. However, during the last decades there has been a discussion whether the intake of OCs increases the risk for depressive disorders and worsens symptoms in women already suffering from depression, while other studies show contradictory results^3–5^. The heterogeneity could be due to manifold reasons including “baseline levels of endogenous hormones (…) and variables indicative of differential hormone sensitivity”^3^. Within this heterogeneity a large prospective Danish study recently showed comprehensively that women taking OCs are at higher risk of developing a mood disorder^6^. However, the underlying biological mechanisms are still elusive.

Our study focused on the hypothalamic–pituitary–adrenal axis (HPA-axis) as it was repeatedly shown that OCs increase free circulating cortisol in the blood^7–9^. Chronically elevated cortisol levels and reduced diurnal rhythm are markers of permanent stress^10^ and have been implicated in the aetiology of several mental disorders^10–12^. Furthermore, chronic stress and elevated corticosterone levels were shown to inhibit neurogenesis within the hippocampus in rodents, thus promoting depressive symptoms^13–17^. Hence, we hypothesized that the physiological response to OCs might mimic to some extent those alterations caused by chronic mental stress.

A central regulator of the HPA axis is the F056 binding protein FKBP5. Expression of the corresponding gene *FKBP5* is positively regulated by glucocorticoid receptor (GR) signaling^18–21^. In turn, FKBP5 regulates GR sensitivity by binding the receptor in the cytoplasm and inhibiting its translocation to the nucleus^18,21,22^. Hence, FKBP5 is the central module of a negative feedback loop fine-tuning cortisol action, making it an interesting candidate for the investigation of stress related disorders on the molecular level. Indeed, the minor T allele of the single nucleotide polymorphism (SNP) rs1360780 has been demonstrated to be associated with increased expression of *FKBP5* ^19,20,23,24^, while in combination with childhood abuse it was also shown to be associated with higher depression risk^18,22,23,25^, altered *FKBP5* methylation levels^19,24,26,27^, and with grey matter volume loss especially in the hippocampus^19,28^. The proposed underlying mechanism assumes that the haplotype represented by the T risk allele increases the expression of *FKBP5*, which is paralleled by decreased DNA methylation of the gene^19,26,27^. The clinical meaning of low FKBP5 methylation levels however is not yet fully established^27^.

While the influences of stress on the transcriptome have been researched extensively, the impact of chronic stress on the metabolome has received attention only recently^29,30^. Interestingly, especially phospholipids and sphingomyelins were highlighted to be influenced by chronic stress, with specific phospholipids being directly correlated to circulating corticosterone in rats^30^. Moreover, it was demonstrated that antidepressants affect the lipid metabolism of the murine brain^31^, reducing ceramide levels in the hippocampus. Thus, we hypothesized that if OCs influence the HPA axis we should observe corresponding alterations in circulating phospholipids in association with OCs as well.

Here, we tested the hypothesis that OCs are associated with stress-like alterations in the FKBP5-GR-signaling circuit in pre-menopausal women from the general population Study of Health in Pomerania (SHIP-TREND-0)^32^, integrating data from multiple biological layers. We aimed at identifying the molecular effects of OCs at the levels of gene expression and metabolomics (including cortisol), *FKBP5* methylation, and structural brain MRI data.

## Results

For comprehensive multi-omics analyses, data from 233 pre-menopausal women aged between 20 and 54 excluding diabetics were available. From these, 74 women (32.2%) used OCs (n = 70 combinations of progesterone and oestrogen, n = 4 progesterone only compounds, see Table 1). The sample description is shown in Table 2. All women taking OCs were experienced users reporting an intake of OCs for at least two years in their lifetime (range 2–35 years) and only 11 women reported having never utilized OCs. Alcohol consumption and smoking were not associated with OC intake, while on a descriptive level, women taking OCs showed less depressive symptoms and a lower childhood trauma questionnaire (CTQ) score than the controls. Thus, to ensure that these differences could not contribute to systematic differences, all analyses were controlled for the psychometric variables in primary or sensitivity analyses. Additionally, we performed propensity score matching to control in a different way for potential confounding caused by psychometric traits (e.g. women with depression may have lower prescription rates for OCs) and other potential confounders^33^.

**Table 1:** Frequencies of used oral contraceptive compounds.

Active Ingredient		Dosage (mg)		N	%
**Single Compound**					
Medroxyprogesteron		92.69		1	1.35
Desogestrel		0.075		3	4.05
**Combined Compound**					
*Monophasic*					
Ethinylestradiol	Levonorgetrel	0.02	0.1	4	5.41
		0.03	0.125	8	10.81
		0.03	0.15	4	5.41
		0.05	0.125	2	2.7
	Desogestrel	0.02	0.15	2	2.7
		0.03	0.15	10	13.51
	Norgestimat	0.035	0.25	3	4.05
	Drespirenon	0.02	3	4	5.41
		0.03	3	3	4.05
	Chlormadinon	0.03	1.79	3	4.05
	Dienogest	0.03	2	15	20.27
*Triphasic*					
Ethinylestradiol	Levonorgetrel	0.03	0.125	2	2.7
		0.04	0.075		
		0.03	0.05		
		0.03	0.125	8	10.81
		0.03	0.05		
		0.04	0.075		
		0.03	0.05	1	1.35
		0.03	0.125		
		0.04	0.075		
Ethinylestradiol	Desogestral	0.03	0.1	1	1.35
		0.03	0.15		
		0.035	0.05

**Table 2:** Descriptive Statistics for the analysed 233 pre-menopausal women of SHIP-TREND-0.

	no OC usage	OC usage	p-Value
N	159	74	
Age (years)	40.1 (7.3)	36.3 (8.7)	0.001^c^
Waist Circumference (cm)	81.0 (11.2)	77.6 (12.1)	0.040^c^
Triglycerides (mmol/l)	1.1 (0.6)	1.4 (0.7)	0.002^c^
rs1360780 (TT carrier, %)	9.43	12.16	0.499^d^
CTQ^a^	33.5 (11.0)	30.5 (8.3)	0.037^c^
BDI-II^b^	9.7 (7.0)	7.0 (3.9)	<0.001c
Lifetime depression (%)	24.05	14.86	0.123^d^
Alcohol Consumption (g/day averaged over the last 30 days)	5.5 (0.8)	4.1 (0.5)	0.134^c^
Current Smokers (%)	34.18	24.32	0.170^d^
Time interval of OC intake in whole life (years)	9.7 (0.6)	17.2 (0.9)	<0.001^c^
Time of Blood sampling	9.20 am	9.06 am	0.190^c^
Mean Methylation	0.88 (0.003)	0.89 (0.002)	0.016^c^

^a^CTQ = Childhood Trauma Questionnaire. ^b^BDI-II = Beck Depression Inventory II. ^c^p-values from Welch t-test. ^d^p-values from Fisher’s exact test.

### Evidence for Alterations of the Blood Lipidome in Women Taking OCs

**Figure 1:**
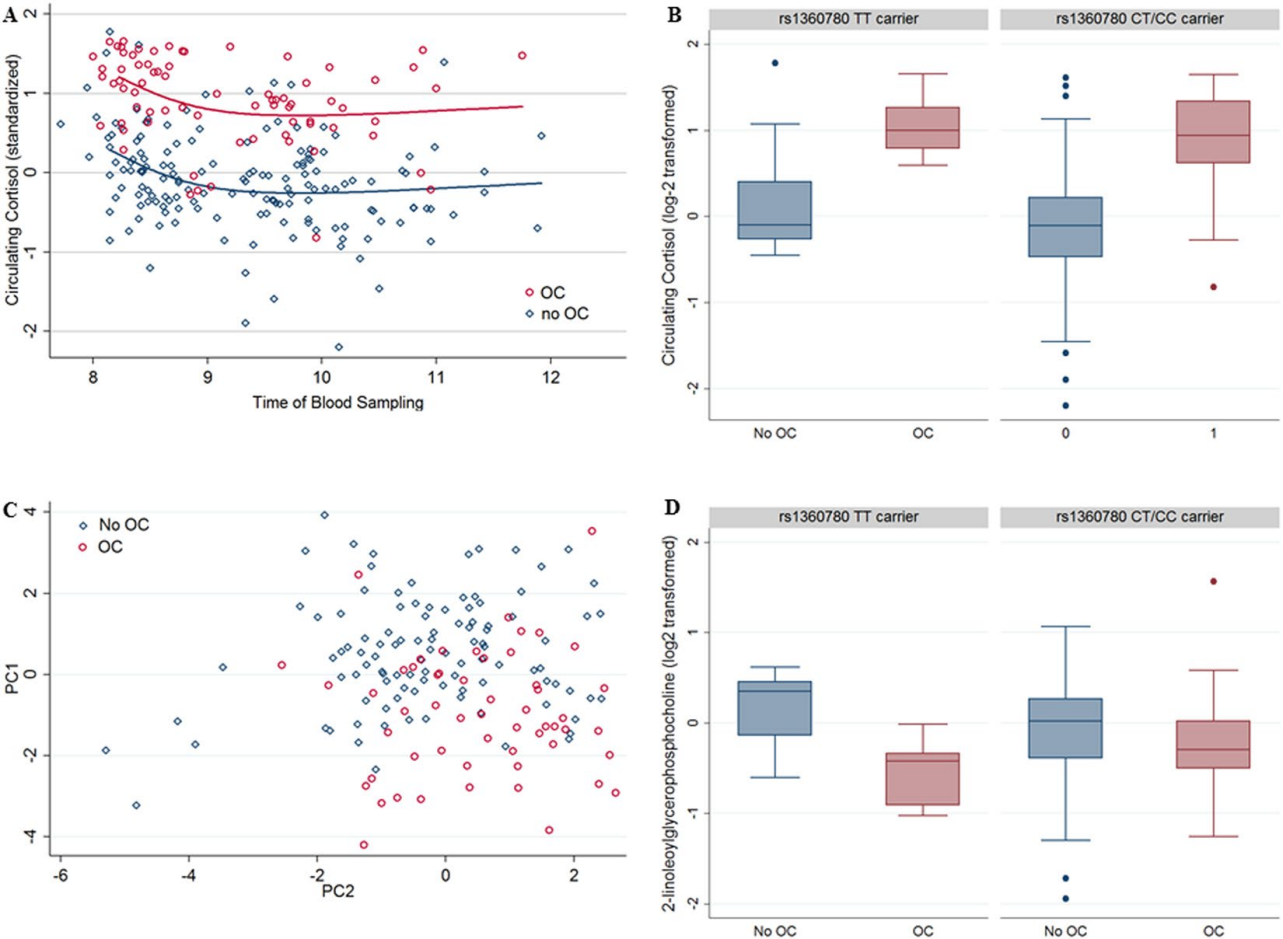
Oral Contraceptives (OC) and metabolites (n = 230). (**A**) Relationship between Cortisol levels (Y-axis) and the time of blood sampling (X-axis) for OC users (red) and non OC users (blue) with non-linear regression line. (**B**) Box plots for circulating cortisol levels stratified for OC use and rs1360780 genotype. The main effect of OC intake (b = 0.92 95%-(CI): (0.75; 1.09), p = 6.734e-22) is not significantly modified by the rs1360780 genotype (b = 0.24, 95%-CI: (−0.18; 0.66), p = 0.259). (**C**) Scatter plot of the first two principle components (PCs) of the significantly associated phospholipids for OC users (red) and non OC users (blue) (**D**) Box plots for 2-Linoleoylglycerophosphocholine levels stratified for OC use and rs1360780 genotype. The main effect of OC intake (b = −0.24,95%-CI: (−0.39; −0.10), p = 0.001) is modified by the rs1360780 genotype (b = 0.63, 95%-CI:(0.26; 1.01), p = 0.001).

Multivariable regression analyses adjusted for the time of blood sampling (range: 8–12 AM), fasting time, age, waist circumference, and blood cell counts detected a significant association between the usage of OCs and higher circulating cortisol levels (b = 0.92 95%-confidence interval (CI): (0.75; 1.09), p = 6.734e-22), see Fig. 1A and B. As additional analyses, we performed propensity score matching, but the result regarding the association of OCs with cortisol remain stable with a slightly higher effect size (b = 1.01, 95%-CI:(0.83; 1.21), p = 5.93e-26). The effect size was sufficient to classify OC users from non-OC users exclusively based on their blood cortisol level with an area under curve (AUC) of 0.91 (95%-CI: (0.86; 0.96), p < 0.0001, see supplementary Fig. S1). OC usage contributed 39.4% of variance to the circulating cortisol levels and was thus far more important in terms of variance than the time of blood sampling (6.46% variance contribution). Interestingly, the time span of using OCs in whole life was not significantly associated with cortisol levels (b = −0.00, 95%-CI: (−0.1; 0.1), p = 0.677). In analogous regressions, OC intake was associated with different levels of numerous lysoglycerophospholipids (9 from 41 tested species, see Table 3) as well as with elevated blood triglyceride levels. As shown in Fig. 1C representing the scatter plot of the first two principle components (for details of the principle component analyses, see Tables S1 and S2) of the significant phospholipids, the effects of OC intake on the phospholipidome were also strong enough to allow significant classification (AUC:0.85, 95%-CI: 0.79; 0.92, p < 0.0001). In analogy to earlier studies, these effects were partly or completely mediated by cortisol^34,35^ and consequently all the OC-related lipid species showed a significant association to circulation cortisol, adjusted for the same covariates as before (see Table 3). Interestingly, for one of these phospholipids, namely 2-linoleoylglycerophosphocholine, the OC effect was modified by the rs1360780 genotype where homozygote risk allele (TT) carriers taking OCs exhibited a stronger blood level decrease (interaction effect: b = 0.63, 95%-CI:(0.26; 1.01), p = 0.001, see Fig. 1D) as compared to the other genotype carriers. This effect modulation was not observable for cortisol (b = 0.24, 95%-CI: (−0.18; 0.66), p = 0.259, see Fig. 1B). In sensitivity analyses, neither the inclusion of alcohol intake, smoking, CTQ scores nor the inclusion of current depressive symptoms (Beck Depression Inventory-II, BDI-II), or the diagnoses of a lifetime major depressive disorder (MDD) as covariates altered these associations substantially (see Supplementary Table S3).

**Table 3:** Association of different phospholipid species and total triglycerides with OC usage and cortisol.

Compound	OC usage					cortisol		p-value for mediation^d^
	Model 1^a^			Model 2^b^		Model 3^a^		
	Mis., %	b(95%-CI) for OC usage	p^c^	b(95%-CI) for OC usage	p^c^	b(95%-CI) for cortisol	p^c^	
Triglycerides	1.7	0.37(0.18; 0.57)	0.0001	0.27(0.06; 0.48)	0.0129	0.22(0.11; 0.34)	0.0001	0.020
1-linoleoylglycerophosphocholine (18:2n6)	0.4	−0.23(−0.37; −0.10)	0.0009	−0.17(−0.33; 0.00)	0.0548	−0.14(−0.24; −0.04)	0.006	0.122
1-palmitoylglycerophosphate	8.3	0.36(0.17; 0.56)	0.0002	0.16(−0.06; 0.38)	0.1425	0.28(0.16; 0.39)	2.26e-06	0.001
2-linoleoylglycerophosphocholine	0.4	−0.24(−0.39; −0.10)	0.0011	−0.14(−0.32; 0.05)	0.1539	−0.17(−0.27; −0.08)	0.0004	0.029
1-palmitoylglycerophosphoinositol	17.0	0.44(0.30; 0.59)	5.99e-09	0.29(0.08; 0.49)	0.0061	0.29(0.20; 0.38)	1.30e-09	0.007
1-palmitoylglycerophosphoethanolamine	0.4	0.32(0.19; 0.44)	1.08e-06	0.15(0.00; 0.29)	0.0486	0.24(0.15; 0.33)	1.62e-07	0.004
2-palmitoylglycerophosphoethanolamine	20.9	0.30(0.14; 0.46)	0.0002	0.13(−0.07; 0.33)	0.1969	0.24(0.13; 0.33)	1.12e-05	0.001
1-oleoylglycerophosphoinositol	9.6	0.36(0.18; 0.54)	0.0001	0.24(0.02; 0.46)	0.0350	0.23(0.11; 0.35)	0.0002	0.045
1-docosapentaenoylglycerophosphocholine (22:5n3)	1.3	−0.41(−0.60; −0.22)	3.88e-05	−0.28(−0.51; −0.05)	0.0170	−0.25(−0.38; −0.12)	0.0001	0.033
1-docosahexaenoylglycerophosphoethanolamine	0.4	0.24(0.12; 0.35)	5.05e-05	0.12(−0.02; 0.26)	0.0812	0.17(0.10; 0.25)	7.65e-06	0.005

^a^Model 1: adjusted for age, waist circumference, blood cell counts, fasting time and time of blood sampling. ^b^Model 2: adjusted for age, waist circumference, blood cell counts, fasting time, time of blood sampling and cortisol. ^c^Uncorrected p-values; Bonferroni threshold = 0.0012 (correcting for 41 test). ^d^Bootstrap derived p-value using 1000 replications for the significance of the indirect effect (difference between regression weights of OC intake between model 1 and model 2).

### Evidence for Higher Genomic GR signalling in Women taking OCs

**Figure 2:**
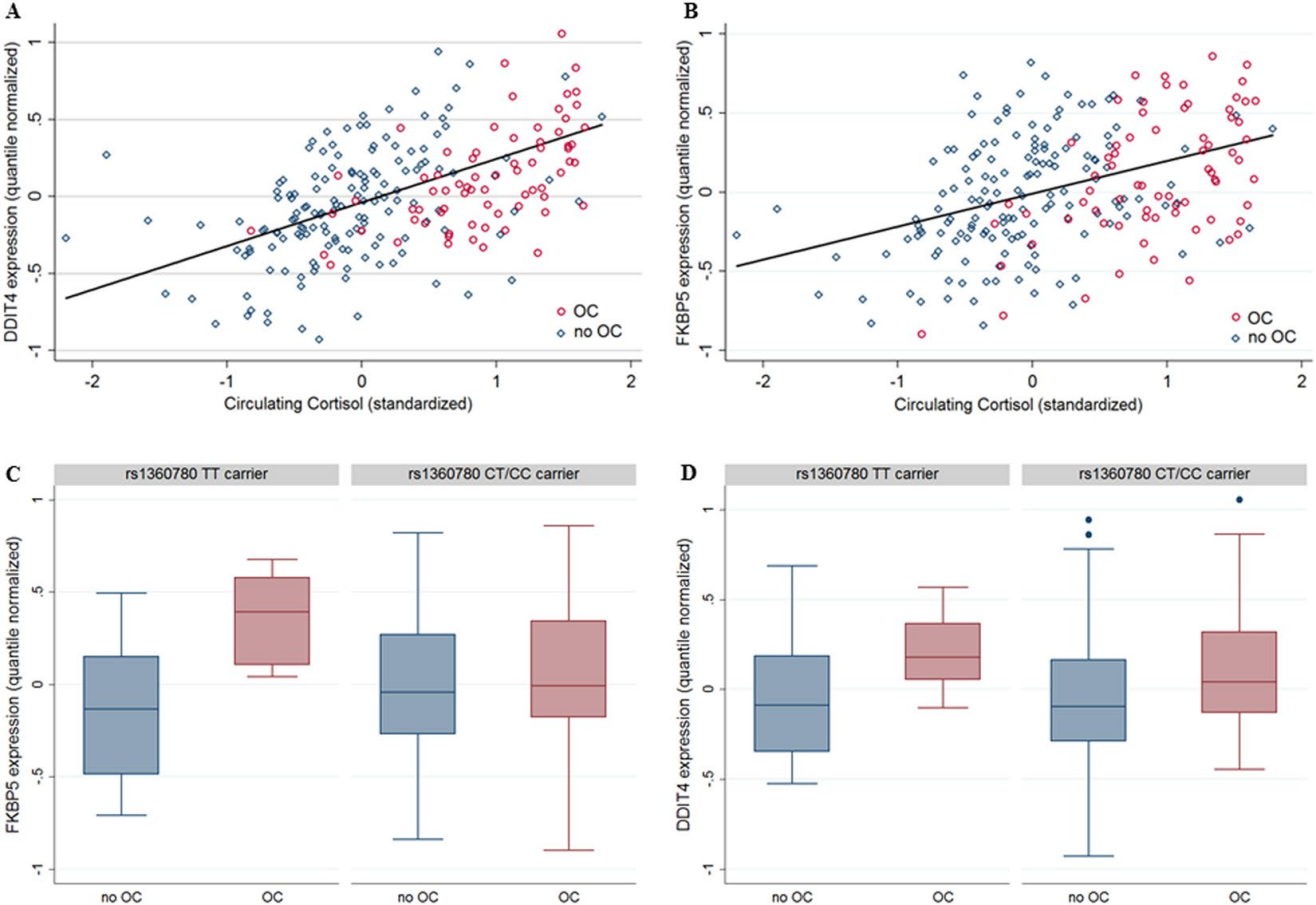
Oral Contraceptives (OC) and Glucocorticoid induced whole-blood transcript levels (n = 226). (**A**) *DDIT4* transcript levels (quantile normalized residuals) against circulating cortisol levels for OC users (red) and no OC user (blue) (**B**) *FKBP5* transcript levels (quantile normalized residuals) against circulating cortisol levels for OC users (red) and no OC user (blue) (**C**) *FKBP5* transcript levels stratified for OC usage and rs1360780 genotype. An interaction between genotype and OC usage was observable (b = −0.34, 95%-CI: (−0.61; −0.08), p = 0.011). (**D**) *DDIT4* transcript levels stratified for OC usage and rs1360780 genotype. No interaction between genotype and OC usage was observable (b = −0.01, 95%-CI: (−0.25; 0.23), p = 0.962).

To test whether the drastically elevated cortisol levels result in enhanced genomic glucocorticoid signalling, we analysed the whole-blood transcript levels of *FKBP5* which is known to be glucocorticoid-induced^18–21,36^. The association analyses was run on the quantile-normalized residuals of the transcripts after correcting for technical covariates, age, sex, waist circumference, blood cell counts, haematocrit, time of blood sampling and fasting time. Indeed, the *FKBP5* mRNA levels were found to be robustly correlated with those of circulating cortisol (r = 0.40, p < 0.0001, see Fig. 2B). Accordingly, the transcript was upregulated in women taking OCs OC-intake (b = 0.11, 95%-CI: (0.00; 0.21), p = 0.045). To test whether this effect is restricted to *FKBP5* or reflects a generally increased GR signalling, we analysed *DDIT4* which has not only been shown to be induced by cortisol^36^, but also represented the top hit of our own transcriptome-wide association study on circulating cortisol (see Supplementary Table S4). Consistently, *DDIT4* mRNA levels were also highly correlated with those of cortisol (r = 0.51, p < 0.0001, see Fig. 2A). In line with increased GR signalling in association with OC intake, the mRNA levels of *DDIT4* were higher in women taking OCs (b = 0.18, 95%-CI: (0.08; 0.27), p < 0.001).

Analysing possible effect modulation caused by the *FKBP5* SNP rs1360780, the effect of OCs on *FKBP5* transcript levels was only present in women representing homozygous TT risk allele carriers (see Fig. 2C) as validated by a statistically significant interaction between OC taking and the rs1360780 genotype on the *FKBP5* transcript amounts (b = −0.34, 95%-CI: (−0.61; −0.08), p = 0.011). In contrast, an interaction with the rs1360780 genotype could not be demonstrated for the *DDIT4* transcript levels (b = −0.01, 95%-CI: (−0.25; 0.23), p = 0.962, see Fig. 2D). Including the depression variables as covariates did not alter the associations; details on sensitivity analyses can be found in the Supplementary Table S5.

### Evidence for altered *FKBP5* Intron 7 Methylation

**Figure 3:**
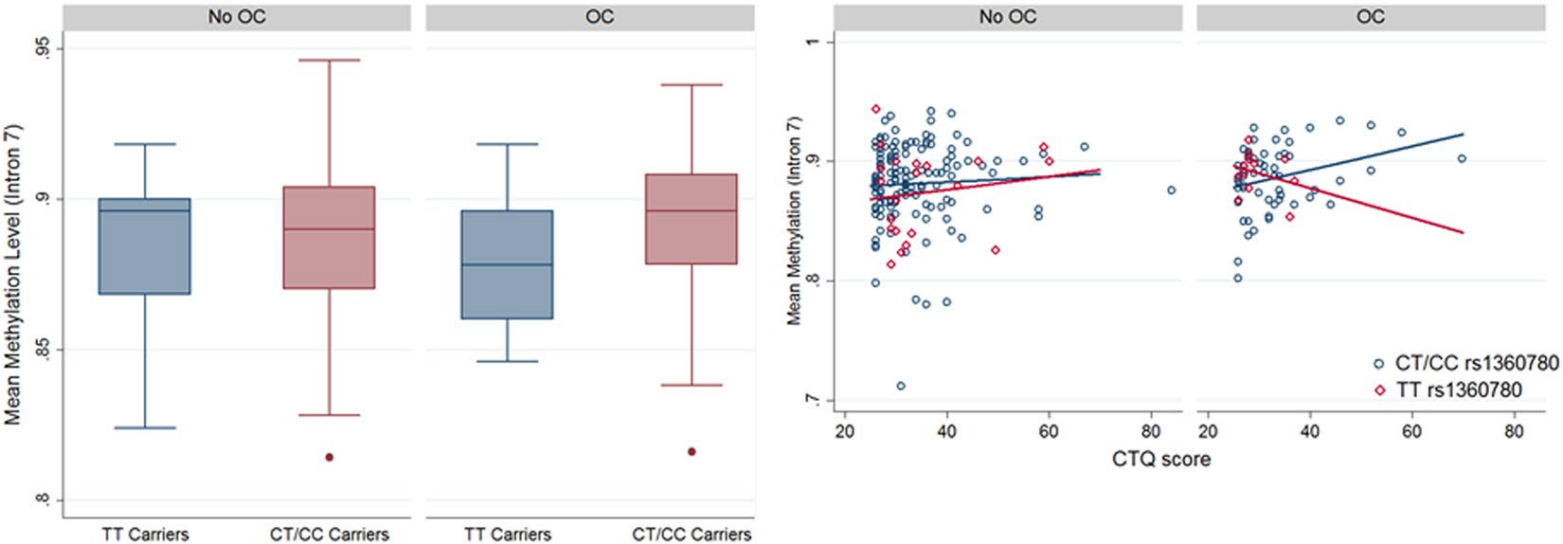
OC intake and mean DNA methylation levels on five CpG sites in intron 7 of *FKBP5*. (**A**) Boxplots stratified for OC intake and the rs1360780 genotype. The effects of OC taking are modified by the rs1360780 genotype (OR = 1.23, 95%-CI: 1.00–1.53, p = 0 0.045). (**B**) Scatter plots CTQ score vs DNA methylation levels with corresponding regression lines stratified for OC intake and rs1360780 genotype (TT carrier red; GT/GG carrier blue). The regression lines in tendency do not have the same slope (OR = 1.03, 95%-CI: (1.00; 1.06), p = 0.074).

Based on the reported observation that the rs1360780 genotype in combination with childhood trauma is associated with the methylation of certain CpG sites in intron 7 of *FKBP5* ^19,24,26,27^ we tested for similar effects regarding the usage of OCs on these methylation sites. The mean detected methylation over the five analysed CpG sites indeed demonstrated the predicted interaction effect between taking OCs and the rs1360780 genotype (OR = 1.24, 95%-CI: (1.00; 1.53), p = 0.045; see Fig. 3a), where homozygous risk allele carriers taking OCs exhibited the lowest methylation. This effect did not change after including cortisol, depressive symptoms, MDD and the CTQ score as covariates (see Supplement Table S6). The analyses were adjusted for time of blood sampling, fasting time, age, waist circumference and blood cell counts. Note that we ran a semiparametric regression methodology^37^ developed especially for fractional data (like methylation data) which avoids some of the problems of beta regression or logit transformations.

We were not able to replicate this result on methylation in a *second independent* SHIP cohort (SHIP-2, n = 300 pre-menopausal women with methylation data). The SHIP-2 cohort, however, consisted of considerably older women (aged 31–55, see Supplementary Table S7) and the sampling was not controlled for fasting-time (mean fasting time in SHIP-2 was 4.5 h; in SHIP-TREND 10.8 h), so the analyses were not completely comparable. Nevertheless, we detected an interaction between CTQ scores and OC intake on methylation levels (OR = 1.01, 95%-CI:1.00–1.01, p = 0.003) in the combined analyses of both cohorts. Whereas in women not taking OCs the CTQ scores were not associated with methylation levels, these were positively correlated to the CTQ scores in women taking OCs (see Fig. 3b). Additionally, the rs1360780 genotype in tendency modified the association between CTQ scores, OC intake and the mean methylation levels in *FKBP5* intron 7 (OR = 1.03; 95%-CI: (1.00; 1.06), p = 0.074, see Fig. 3b). For women taking OCs, we observed that with higher CTQ scores methylation levels decreased in the homozygous TT risk allele carriers whereas in carriers of the other two genotypes the opposite was the case. Again, these results were robust against the inclusion of depression variables as covariates (see Supplementary Table S8).

### Evidence for Effect Modification of OC Taking on the Association between Cortisol and Depressive Symptoms

**Figure 4:**
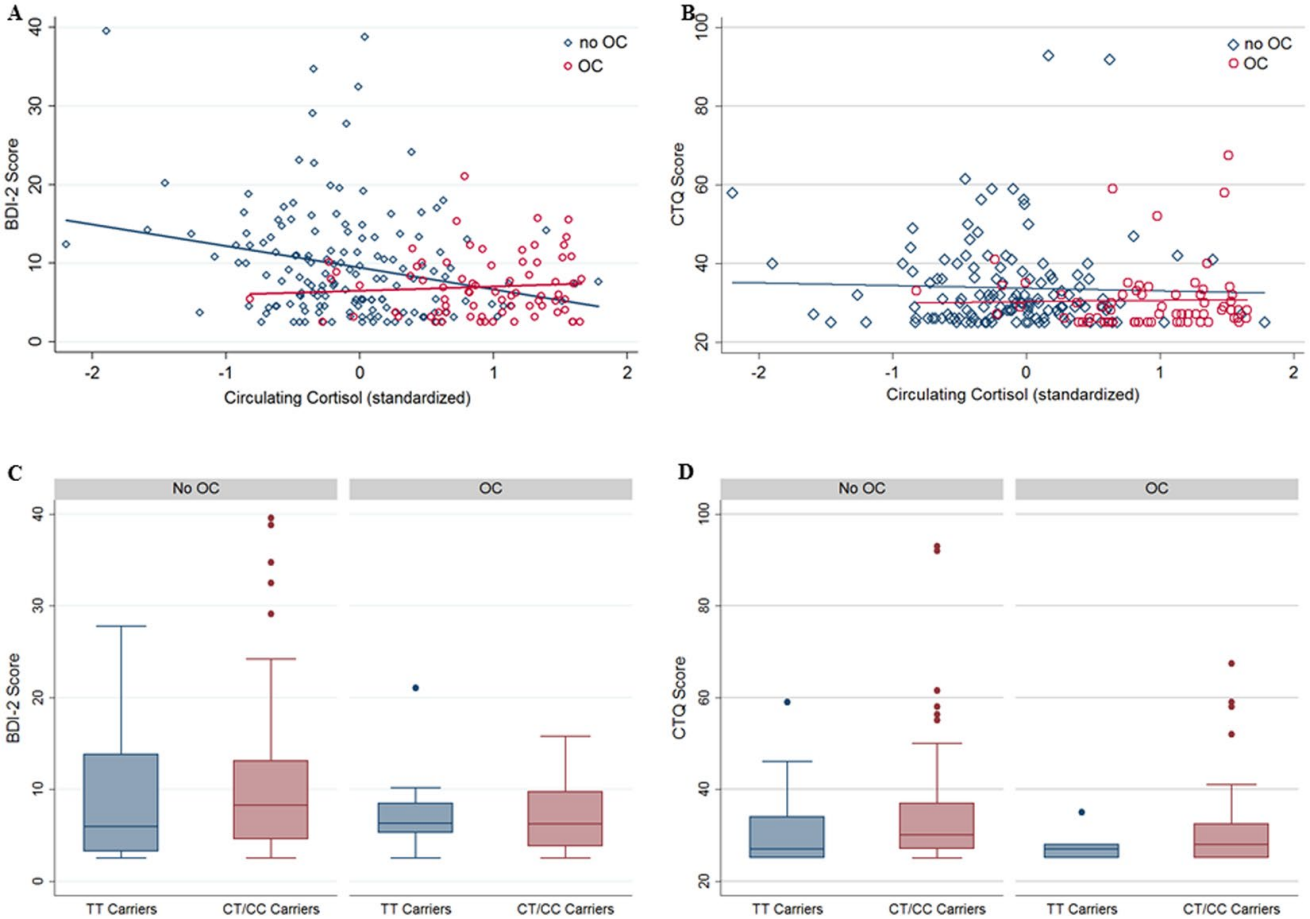
OC intake and clinical questionnaire scores (n = 230 for BDI-II, n = 200 for CTQ). (**A**) Scatter plot BDI-II scores (y-axis) vs. circulating cortisol levels (X-axis) with corresponding regression lines stratified for OC intake. The slopes of the regression lines were significantly different (b = 2.98, 95%-CI: (0.32; 5.64), p = 0.028). (**B**) Box plots for BDI-II scores stratified for OC intake and rs1360780 genotype with no interaction present between OC intake and the rs1360780 genotype (b = −0.05, 95%-CI: (−5.09; 4.98), p = 0.984). (**C**) Scatter plot CTQ scores (y-axis) vs. circulating cortisol levels (X-axis) with corresponding regression lines stratified for OC intake. The slopes of the regression lines were not significantly different (b = 1.01, 95%-CI: (−4.12; 6.14), p = 0.699). (**D**) Box plots for CTQ scores stratified for OC intake and rs1360780 genotype with no interaction present between OC intake and the rs1360780 genotype (b = 0.31, 95%-CI: (−7.06; 7.68), p = 0.934).

After detecting cortisol-related and partly genotype-dependent alterations of HPA-axis signalling mediated by OC usage on different omics levels (epigenomic, transcriptomic and metabolomic), we turned our attention to the clinical phenotype of depressive symptoms. Using the BDI-II score as outcome parameter, we fitted a multivariable regression (controlling for age, waist circumference, CTQ and time of blood sampling) investigating the association of circulating cortisol and OCs with depressive symptoms. Respecting the non-Gaussian distribution of the BDI-II scores, the reported CIs were derived by bootstrapping with 2000 repetitions. As shown in Fig. 4A the association between circulating cortisol and depressive symptoms clearly depended on OC usage. Whereas in non-OC users low cortisol levels were associated with a higher load of depressive symptoms, no association between cortisol levels and depressive symptoms was found in OC using women (see Fig. 4A). Accordingly, we detected a corresponding interaction effect in the above mentioned model (b = 2.98, 95%-CI: (0.32; 5.64), p = 0.028). There was no interaction between OC intake and the rs1360780 genotype regarding depressive symptoms (b = −0.05, 95%-CI: (−5.09; 4.98), p = 0.984, see Fig. 4B). We did not observe any interaction of cortisol with OC intake (b = 1.01, 95%-CI: (−4.12; 6.14), p = 0.699) or the rs1360780 genotype with OC taking regarding the CTQ score either (b = 0.31, 95%-CI: (−7.06; 7.68), p = 0.934, see Fig. 4C,D), making a possible confounding by childhood trauma implausible.

### Evidence for Reduced Hippocampal Volumes in OC consuming women

As elevated cortisol levels have been reported to inhibit neurogenesis in the murine hippocampus^13,38,39^, we tested whether hippocampus volumes as determined by structural MRI data differed between OC using women and those not taking OCs. Adjusting for age, the intracranial volume, the waist circumference, the CTQ score, the BDI-II score and the years of education, we found a negative association between OC usage and hippocampus volume, averaged over both hemispheres (b = −99.31, 95%-CI: (−182.36; −16.26), p = 0.019). Propensity score matching using the same variables as predictors supported the result by giving an equally significant effect for OC intake (b = −87.28, 95%-CI: (−170.86; −3.71), p = 0.041). In sensitivity regression analyses, we additionally adjusted for cortisol, but the effect remained significant (b = −179.47, 95%-CI: (−285.74; −73.19), p = 0.001, see Table 4). However, further adjustment for triglycerides led to a partial mediation of the effect (b = −71.8, 95%-CI: (−155.6; 11.8), p = 0.070, see Table 4), indicating that the increase in triglycerides in association with OC intake might be partially responsible (in a statistical sense) for the effects seen in the hippocampus. We did not find any evidence for an effect moderation of the SNP rs1360780 (see Supplementary Table S9). Full results on sensitivity analyses regarding different covariates can be found in Supplementary Table S9.

**Table 4:** Association of OC intake with Hippocampal Volumes.

	N	Model 1^a^	P	Model 2^b^	p	Model 3^c^	p
		b(95-% Ci)		b(95-% Ci)		b(95-% Ci)	
**Hippocampus**							
SHIP-TREND	185	−99.3 (−182.4; −16.3)	0.019	−179.5 (−285.7; −73.2)	0.001	−71.8 (−155.6; 11.8)	0.090
SHIP-2	143	−48.0 (−148.2; 52.2)	0.345	—	—	−44.1 (−143.6; 55.3)	0.381
Combined^d^	328	−78.2 (−141.4; −14.9)	0.016	—	—	−63.2 (−125.8; −0.7)	0.048

^a^Model 1: adjusted for intracranial volume, age, waist circumference, CTQ score, BDI-II score and education. ^b^Model 2: adjusted for intracranial volume, age, waist circumference, CTQ score, BDI-II score, education and circulating cortisol. ^c^Model 2: adjusted for intracranial volume, age, waist circumference, CTQ score, BDI-II score, education and blood triglycerides. ^d^Combined analyses with additional adjustment for cohort.

Finally, we tried to replicate the MRI results in the independent SHIP-2 cohort. For the hippocampus in regression analyses, we found an effect in the same direction missing significance (b = −48.00, 95%-CI: (−148.21; 52.22), p = 0.345). However, propensity score matching in SHIP-2 analogous to the SHIP-TREND analyses revealed a strong negative, significant effect (b = −128.60, 95%-CI: (−242.26; −19.5), p = 0.027), suggesting that in SHIP-2 the primary regression analyses may be biased. The combined regression analysis on both samples revealed an overall significant effect for OC intake (b = −78.15,95%-CI: (−141.43; −14.88), p = 0.016) which was once again diminished by the inclusion of triglycerides (see Table 4). Thus, replicable in two independent samples, we found that women taking OCs displayed smaller hippocampal volumes.

## Discussion

OCs, being an integral part of birth control all over the world^2^, exhibit complex effects on the physiology of women. Although being investigated for several decades, the effects and their possible pathophysiological implications for the aetiology of mental disorders are still not well understood. However, recent data from Danish registers implicate that there is an imperative to understand the actions of OCs for a safe and convenient application as women taking oral contraceptives were described to be at higher risk of incident depression^6^. The study presented here aimed to improve the understanding of the physiological alterations in the HPA-axis in association with the intake of OCs.

Our results reveal not only clearly elevated circulating cortisol levels in women taking OCs, but also some of the metabolic consequences of increased cortisol signalling: Triglyceride levels were increased and we found several phospholipids (Table 3) to be differentially associated with OC intake. Our data illustrate that these metabolite alterations were mediated by the OC-induced increase in cortisol. Earlier studies in rats demonstrated an altered phospholipid profile in the brain of the animals exposed to chronic unpredicted stress. As these alterations were related to higher circulating corticosterone levels^29,30^, our results fit well with these findings from animal studies, because we could demonstrate broad associations between phospholipids and cortisol. Hence, cortisol signalling is likely related to circulating phospholipids in humans, resulting in a stress-related signature in the human lipidome. The distinct role and effect sizes of the found OC lipid associations have to be subject to further investigation. In our data several lysophospholipid species were downregulated while others being upregulated seemingly depending on the chain length, indicating that the picture might be more complex than initially anticipated. Moreover, because of the chosen metabolomic platform, our results consisting only of lysophospholipids cannot deliver the full picture of the phospho-lipidomic alterations associated with OC taking, limiting the interpretability. A pharmaco-metabonomic design would be necessary to clarify these effects conclusively. However, the results provided here are still informative, especially as we could demonstrate that OC taking influences blood metabolite levels dependent on the rs1360780 genotype.

Consistently, the whole blood transcriptome data showed that the transcript levels of *DDIT4* and *FKBP5*, both known to be positively regulated by GR^20,21,36^, were increased in women taking OCs. In the case of *FKBP5*, encoding a potent inhibitor of glucocorticoid signalling as part of a negative regulatory feedback loop^18,21^, we observed an effect modulation by the *FKBP5* SNP rs1360780 which was also observed in the metabolome data.

This finding mirrors earlier association findings on rs1360780 in combination with childhood trauma^19,20,22–26,40^. In extension of the hypothesis presented by Klengel *et al*.^19^, we propose that every condition leading to strong cortisol level elevations in combination with the presence of the homozygous TT risk allele of rs1360780 might result in increased *FKBP5* expression. This could be mediated by an altered accessibility of the *FKBP5* DNA for methyltransferases specified by the underlying haplotype^19,26,27^. Coming back to our initial hypothesis that the effects of the elevated cortisol levels in association with OCs mimic the effects of chronic stress, this is exactly what we see in association with the usage of OCs in our data.

The effect was also observable at the level of *FKBP5* DNA methylation in intron 7, displaying essentially the same results reported in studies regarding the interaction of traumatic experiences with the rs1360780 genotypes^19,24,26,27,41^. Similar to these studies which reported that individuals having experienced trauma and being rs1360780 TT risk genotype carrier’s exhibit decreased *FKBP5* methylation, women with that genotype and taking OCs showed the lowest *FKBP5* methylation. Furthermore, the relation of childhood trauma and methylation level was again modified by OC intake in a genotype-dependent way.

According to our results, alterations of the HPA axis caused by OC intake depend on the individual history as well as the genetic background. Thus, it is plausible to assume that the risk for OC-intake related depression is modified by these factors, too. Women at specific high risk for the development of a mood disorder when taking OCs might therefore be identifiable by SNP typing of rs1360780 and by requesting information on possible traumatic experiences. Naturally, as our design was cross-sectional in nature, this aspect is left for future research.

Regarding current depressive symptoms, we observed a striking interaction between circulating cortisol and OC usage with inverted effect signs for cortisol in OC users and OC non-users. This is clearly of methodological relevance, because the question whether higher cortisol levels are actually associated with depression is inconclusive and intensively discussed^40,42^. However, as most studies were conducted in industrialized societies with high prevalence of OC intake, the usage of OCs could be an important confounding and moderating factor in this debate. Our results suggest that studies should stratify according to OC usage or, at least, consider OC usage as a covariate, carefully checking plausible interactions. Our study failed to deliver an explanation for this interaction as a plausible candidate within our framework, the SNP rs1360780, showed no effect in combination with OC intake regarding depressive symptoms. Nevertheless, FKBP5 levels were enhanced in OC users which might compensate the impact of enhanced cortisol levels. Revealing the mechanism in behind the reported interaction between circulating cortisol and OC usage is clearly a valuable topic for future research. Being cross-sectional in nature, our study (finding that women using OCs showed less depressive symptoms) does not contradict the results of Skovlund *et al*.^6^. First, as age was shown to influence the effects of OC usage on depression^3,4,6^, it may be that the in comparison to earlier study higher age of study participants here contributed to the lack of evidence for a direct association between OC usage and depression. Studies supporting the hypothesis of OC usage enhancing a depressive risk are mainly based on adolescents or young adults^3–6^. Most importantly, the cross-sectional design of our study may have led to selection bias known as “survivor effect” as it is plausible that women taking OCs in our study are those who did not experience the negative side effects on mood^3,43^. This might account for the observed difference in BDI scores between women taking OCs and controls. Note, however, that the goal here was not to deliver evidence for OC usage being a risk factor for the development of a depression, but to examine the consequences of OCs on the HPA axis.

Because several studies related chronic stress and elevated cortisol levels to decreased neurogenesis in the hippocampus^13,38,39^ we tested if OCs might be associated with structural changes in the brain using MRI scan data. Indeed, we detected a reduction of hippocampal grey matter associated with the intake of OCs in two independent samples. This main finding was importantly robust in propensity score matching analyses, indicating that it was not an artefact caused by depression related selection bias. The effect was unexpectedly not mediated by the higher cortisol levels at least from a statistical viewpoint. Having this said, the effects of OCs on brain structure are in need of a mechanistic explanation, but it may be that the underlying physiological factor is related to the observed alterations in the lipidomic profile associated with OC intake. In our modelling, the inclusion of blood triglyceride levels attenuated the effect sizes of OC intake, indicating effect mediation. Keeping in mind that obesity, a main factor for elevated triglycerides, is associated with a global reduction of grey matter volume^44,45^, the plasma lipidome may be an important factor in neurodegenerative processes^46^.

Despite integrating several different categories of omics data, our study also has several limitations. First of all, our design was purely observational and thus cannot establish causality. We describe coherent alterations associated with OC intake for different systemic levels, but we cannot clearly demonstrate a causal role for OCs in the absence of experimental data. For the same reason, we cannot link the different Omics layers to each other via a causal model in a strict sense, although the mediation analyses suggests that the driving factor in behind of the metabolic and transcriptomic associations is the raise in circulating cortisol. We cannot rule out the possibility for unmeasured confounding, despite adequate layer specific covariate inclusions. However, it should be kept in mind that a potential confounder would have to modify the probability of OC intake as well as the dynamics of the HPA axis. One plausible candidate for such a confounder would be depression itself, but our analyses showed that the associations of OC intake with HPA-axis alterations were consistently independent from depressive symptoms. Moreover, we found the stress related signatures despite the fact that the women taking OCs were slightly less depressed than the controls, making a confounding by this factor non-plausible.

Another import limitation related to the cross-sectional design is given by a potentially present survivor effect as already mentioned. The non-user group may include women who once used OCs stopped the intake due to negative side effects on mood. These side effects could be associated with a major HPA-axis deregulation resulting, for example with enhanced cortisol levels. If this would be the case, our effect sizes might even underestimate the real effects. Propensity score matching suggested indeed that for cortisol the true effect may be slightly larger than indicated by classical regression methodology. The selection bias may be more drastic on MRI results where selection represents a severe problem as individuals with psychiatric disorders may tend to avoid MRI scans. Our results were stable in the light of propensity score matching, but prospective studies are necessary to validate the implications of our study. One further limitation could be seen in the fact that the time of blood sampling varied between 8–12am. We used the time variable throughout the analyses as a covariate making it unlikely that this variable adds to the systematic variation between cases and controls. However, given our results, it may be possible that oral contraceptives may interfere with the circadian rhythm, resulting in a shift of the morning rather than a permanent increase. This could only be clarified in a corresponding time-series design.

In general, our results fit well to earlier findings, but some of the findings, especially the methylation evidence, require additional validation. Moreover, the number of rs1360780 risk allele carriers was relatively small in our study sample, which may limits generalizability. Additionally, we were not able to differentiate between specific types of OCs because of the small case numbers or to adjust for different free gonadal hormone levels which may be an important mediating variable. Therefore, we cannot evaluate the impact of the concrete type of OC. The exact duration of OC intake may be also an important covariate which should be investigated more closely. It should be noted that our results do not generalize necessarily to other hormonal contraceptives like implants or patches due to the different pharmacokinetics of the application. Nevertheless, the well-founded hypothesis that OCs may cause substantial stress-like alterations in the HPA axis on different physiological levels and may lead to grey matter losses in the hippocampus is a serious concern which demands further research to foster a safe and convenient application of OCs.

## Methods

### Study Population

We analysed data from the Study of Health in Pomerania (SHIP) comprising adult German residents from north-eastern Germany^32^. A two-stage stratified cluster sample of adults aged 20–79 years (baseline) was randomly drawn from local registries. The SHIP-TREND-0 cohort, from which a random sample of 1000 individuals excluding diabetics was selected for comprehensive multi-omics characterization, was originally recruited 2008–2011 (n = 4420). Using a “women’s questionnaire” including the question “Do you still menstruate?” we selected women answering the question in the affirmative as pre-menopausal. The subsample used in the present study included 233 pre-menopausal women (age range: 20–54 years) with available genome-wide SNP typing as well as whole-blood methylation data. For 230, 226, and 196 women, valid blood cortisol measurements, whole-blood transcriptome data, and brain MRI scans for volumetric analyses were available, respectively.

For replication of the brain MRI- and the methylation results of SHIP-TREND, a second independent SHIP sample, was used: SHIP-2 (n = 2333, 2008–2012) is the ten years follow-up of the baseline SHIP-0 sample, recruited from the same area as SHIP-TREND-0. Note that SHIP-2 and SHIP-TREND-0 are without overlap and sampled independently of each other. For 1163 SHIP-2 participants including 150 pre-menopausal women, selected as specified for SHIP-TREND-0, aged 30–58, brain MRI scans were available. For 303 pre-menopausal women, whole-blood DNA methylation data were collected.

All analyses were performed in accordance with the Declaration of Helsinki, including written informed consent of all participants. The survey and methods of the SHIP studies were approved by the institutional review boards of the University of Greifswald.

### Interview and psychometric data

Sociodemographic factors and medical history were assessed by a computer-assisted face-to-face interview. Current depressive symptoms were assessed using the Beck Depression Inventory-II (BDI-II) in SHIP-2, which is a 21-item self-report questionnaire with high reliability and validity^47^. In SHIP-TREND-0 current depressive symptoms were assessed by the PHQ-9. To optimize the comparability of both assessments the PHQ-9 score was transformed into BDI-II. Wahl *et al*. (2014) describe the methodical background in detail^48^. In short, they applied Item-Response-Theory (IRT) methods to develop an IRT metric for eleven different depression tools including PHQ-9 and BDI-II. From that the estimated responses to each of the BDI-II items were added to provide a sum-score. The back-transformation into the PHQ-9 score yielded a correlation of r = 0.98. The diagnoses of any depressive disorders were assessed using the Munich-Composite International Diagnostic Interview (M-CIDI) in SHIP-2 and SHIP-Trend-0^32,49^. The M-CIDI is a standardized fully structured instrument for assessing psychiatric disorders over the lifespan according to DSM-IV criteria. Test-retest reliability analyses of the diagnosis of major depressive disorder (MDD) revealed kappas between 0.62 and 0.77^50^. The Childhood Trauma Questionnaire (CTQ) has a total of 28 items that are rated on a five-point Likert scale, with higher scores indicating a higher exposure to traumatic experiences^51^.

Having completed the interview, participants underwent medical examinations, including the measurements of height and weight to calculate the body mass index (BMI) and waist circumference. The waist circumference was used as covariate in all analyses as it represents a measurement of abdominal fat without being biased by muscle mass like the BMI which may introduce bias when metabolic parameter are analysed. All subjects were informed to bring in their packing containers of all medication they had taken during the last 7 days, as well as their drug prescription sheets. Every compound was recorded and categorized according to the Anatomical Therapeutic Chemical (ATC) classification^52^. All ATC codes beginning G03AA, G03AB and G03AC were classified as OCs. These procedures were equivalent in SHIP-2 and SHIP-TREND.

### Measurements

Glycated haemoglobin (HbA1c) concentrations were determined by high-performance liquid chromatography (Bio-Rad Diamat, Munich, Germany). Triglycerides were determined enzymatically (Siemens Healthcare Diagnostics GmbH, Eschborn, Germany). White blood cell count was measured via the XE 5000 from Sysmex (Sysmex Deutschland GmbH, Norderstedt, Germany). Skilled technical personnel performed all assays according to the manufacturer´s recommendations. Additionally, the laboratory is a participant in official quarterly German external proficiency programs.

### MRI data

All images were obtained from the same scanner (1.5 Tesla Magnetom Avanto; Siemens Medical Solutions, Erlangen, Germany). We used the multiplanar reconstruction T1-weighted axial MRI sequence with the following parameters: 1900 ms repetition time, 3.4 ms echo time, flip angle = 15° and a voxel size of 1.0 × 1.0 × 1.0 mm. After file format conversion from DICOM to NIfTI, the whole pre-processing of the T1-weighted images, the segmentation of the hippocampus was carried out with FreeSurfer v5.1.0 (Cambridge, MA, USA). In quality control, 16 segmentations were assessed as potentially erroneous for the hippocampus. These were excluded from analyses.

### SNP-Typing and Imputation

The SHIP-2 sample was genotyped using the Affymetrix Human SNP Array 6.0. Sample processing and array hybridization was done in accordance with the manufacturer’s standard recommendations. The genetic data analysis workflow was created using the Software InforSense. Genetic data were stored using the database Caché (InterSystems). Genotypes were determined using the Birdseed2 clustering algorithm. For quality control purposes, several control samples where added. On the chip level, only subjects with a genotyping rate on QC probe sets (QC call rate) of at least 86% were included. Finally, all arrays exhibited a sample call rate >92%. The overall genotyping efficiency of the GWA was 98.55%. As the *FKBP5* SNP rs1360780 was not directly typed by the Affymetrix Human SNP Array 6.0, genotype data were derived from standard imputation procedures using the software IMPUTE v0.5.0 based on HapMap II. As a common measure for imputation quality the observed by expected variance ratio was used. This ratio was calculated by dividing the observed variance of the SNP by the expected variance based on the imputed allele frequency and Hardy-Weinberg-Equilibrium. The observed by expected variance ratio of 0.99 indicates a very high imputation quality for rs1360780.

Genotyping of the SHIP-TREND-0 subjects (n = 986) was performed using the Illumina HumanOmni2.5-Quad. DNA from whole blood was prepared using the Gentra Puregene Blood Kit (Qiagen, Hilden, Germany) according to the manufacturer’s protocol. Subsequent sample processing and array hybridization were performed as described by the manufacturer (Illumina) at the Helmholtz Center Munich. The final sample call rate was 99.51%. Imputation of genotypes in the SHIP-TREND-0 cohort was performed with the software IMPUTE v2.1.2.3 against the HapMap II (CEU v22, Build 36) reference panel. The total number of SNPs after imputation and quality control was 3,437,411. Information on the Hardy Weinberg equilibrium and genotype distribution for each platform is given in Supplementary Table S10 (all *P*-values > 0.05).

### Whole-Blood DNA Methylation Data

The methylation state of 5 CpG sites in the intron 7 of *FKBP5* was measured by MALDI-TOF mass spectrometry using EpiTYPER by MassARRAY (Sequenom, San Diego, CA), as previously described^53^. First, 500 ng DNA of each sample was bisulfite converted using the EZ-96 DNA Methylation Kit (Zymo Research, Orange, CA, USA). Primers were designed for one amplicon, covering 5 CpG sites, using the EpiDesigner (Sequenom, San Diego, CA) (Supplementary Table S11). The target region was subsequently amplified using bisulfide-specific primers followed by shrimp alkaline phosphatase (SAP) treatment, and RNAse A cleavage (known as T-cleavage) performed according to the standard protocol (Sequenom EpiTYPER Assay). The PCR product fragments were then cleaned by Resin and spotted on 384 SpectroCHIPs by Nanodispenser. The Chip was analysed by Sequenom Autoflex Mass Spectrometer, and data were extracted using SpectroACHIRE software, and massARRAY EpiTYPER v.1.2 software.

### Analysis of Whole-Blood Transcript Levels

Sample collection and whole-blood RNA preparation were described in detail elsewhere^54^. Briefly, fasting whole-blood samples were collected and stored in PAXgene Blood RNA Tubes (BD). RNA was prepared using the PAXgeneTM Blood miRNA Kit (QIAGEN, Hilden, Germany). Purity and concentration of RNA were determined using a NanoDrop ND-1000 UV-Vis Spectrophotometer (Thermo Scientific). For quality control of RNA samples, all preparations were analysed using a 2100 Bioanalyzer and RNA 6000 Nano Lab Chips (both from Agilent Technologies, Santa Clara, CA, USA) according to the manufacturer’s instructions. Samples exhibiting an RNA integrity number (RIN) less than seven were excluded from further analysis. Whole–blood transcriptome profiling of the samples was performed using the Illumina HumanHT-12 v3 BeadChip array. A detailed workflow for the analysis of Illumina gene expression microarray data was described recently within the MetaXpress consortium^54^. For this study, we extracted exclusively the transcript level data of the cortisol induced genes *FKBP5* and *DDIT4*. While *FKBP5* expression was the real target of our study we used *DDIT4* as a control to test whether we observe effects on the GR signalling pathway in whole or selectively on *FKBP5* expression. *DDIT4* was the top-hit of a transcriptome-wide association analyses on cortisol we performed earlier (for details of this analyses see Supplementary Table S4). As *DDIT4* was previously reported to be cortisol induced^36^ and robustly associated with cortisol in our data, we used *DDIT4* as a proxy for GR signalling. We regressed the quantile normalized and log2 transformed transcript level quantification on technical covariates, blood cell counts, haematocrit, mean platelet volume and age in women and then derived the residual variables. This residual variable was used for association analyses. The SHIP-TREND whole-blood transcriptome dataset is available at GEO (Gene Expression Omnibus) public repository under GSE36382.

### Metabolomic Data

The workflow of the metabolomic data has been described elsewhere in detail^55^. Briefly, two separate LC-MS/MS analytical methods were used as previously published^56^ to obtain a broad metabolite panel in plasma samples in an untargeted manner. Raw ion counts of metabolites were rescaled with the median of each runday to avoid differences caused by daily variations of platform performances. Metabolites were only kept, if a valid estimation (3 observations) of the median within a runday was possible. Afterwards all metabolites were log2-trans-formed. We concentrated on the quantification of cortisol and on phospholipid quantifications. Phospholipid quantifications have been reported to be indicative of chronic stress^36,37^ and neurodegenerative processes^46^. Hence, we concentrated on the quantification of cortisol and on phospholipid quantifications. Note that these quantifications are relative quantifications and no information on absolute concentrations was available.

### Statistical Analyses

For descriptive purposes, metric variables were expressed in means and standard-deviations, categorical variables in proportions. Metric variables were compared for OC users and non OC users with Welch t-tests, categorical variables with Fisher’s exact test. For inference, multivariable regression models were run with the model specification being dependent on the type of dependent variable. To control in a different way for possible selection bias due to the cross-sectional design, we applied additionally propensity score matching with nearest neighbour matching with two matches per observation accounting for the same variables as the corresponding regression models^33^. The results were then compared to each other. In all these models, OC intake was included as predictor and the variable of interest. In an exploratory step, we excluded the progesterone only users (n = 4) from analyses, but found no major differences in the results. Thus, in the final models these cases were included.

Regarding the circulating cortisol and lipidomic data, linear multivariable regression were fitted in which the log2 transformed blood concentration of the species was the dependent variable while controlling for blood cell counts, age, waist circumference, fasting time and the time of blood sampling. Heteroscedastic standard errors were used for the estimation of p-values and confidence intervals. Propensity score matching was performed accordingly for circulating cortisol levels. As 41 compounds were tested, we set the Bonferroni corrected significance threshold to 0.0012. Then, we reran the analyses with the inclusion of cortisol as covariate to test on potential effect mediations by cortisol. Furthermore, in sensitivity analyses we adjusted additionally for current depressive symptoms (BDI-II), MDD lifetime, the CTQ, alcohol intake and smoking. In addition, we ran a principle component analyses over the Bonferroni corrected significant lipidomic variables and tested whether the first two components were able to discriminate between OC users and Non OC users via a logistic regression. We calculated therefore the AUC for this model.

For transcript level analyses, we fitted multivariable linear regression models with heteroscedastic robust standard error with the log2 transformed and quantile normalized residuals of *FKBP5* and *DDIT4* mRNA levels as outcome parameters. As these residuals were already corrected for blood cell counts and age, we adjusted only for waist circumference, time of blood sampling and fasting time. In a second model, we included the rs1360780 genotype and tested the interaction term between rs1360780 and OC intake. The same sensitivity analyses were made as for the metabolomic data.

For the DNA methylation data, we used a semiparametric regression methodology^37^ especially developed for fractional data. This regression methodology only needs the specification of the functional form of the predictive mean which was chosen to be logistic in our case. It does not rely on distributional assumptions and its variance estimators are inherently robust against heteroscedasticity. Thereby, it avoids some of the problems connected to beta regressions or ordinary linear regression with transformed methylation rates^57,58^. The chosen methodology has also the advantage of being easily interpretable as one can derive the regression coefficients in form of odds ratios which can be interpreted in the same way as in logistic regression. The averaged methylation rates were used as dependent variables and all models included once again the time of blood sampling, fasting time, age, waist circumference and blood cell counts as covariates. The factor of interest was the interaction term between the rs1360780 genotype and OC intake. In sensitivity analyses on the SHIP-TREND-0 sample, we adjusted the model further for circulating cortisol, current depressive symptoms, MDD and the CTQ score. For replication in the SHIP-2 cohort, we ran analogous models on the SHIP-2 data. Additionally, we ran the combined analyses including the same covariates using both samples. We checked furthermore possible interactions between the CTQ score, the rs1360780 genotype and OC intake on methylation in the combined analyses. This three-way interaction analyses was not feasible on one of the samples alone because the limited case numbers.

To analyse the association of cortisol to depressive symptoms in dependency on OC intake, we ran a linear multivariable regression model with the BDI-II score as dependent variable. As the BDI-II score displayed a severe non-Gaussian distribution, we derived p-values and confidence intervals by bootstrapping using 2000 replications. The predictor of interest was the interaction term between circulating cortisol and OC intake and the model included the age, waist circumference, childhood trauma and the time of blood sampling as covariates. In a second model we tested the interaction of the rs1360780 genotype with OC intake on depressive symptoms, adjusting for the covariates as before.

The modelling of the volumetric brain MRI data was done *via* multivariable linear regressions using heteroscedastic robust standard errors. The hippocampal volume (averaged over both hemispheres) represented the independent variable and the models were adjusted for intracranial volume, age, waist circumference, CTQ score, BDI-II score and education. The analyses were rerun using propensity score matching methodology accounting for the same covariates. In a second run, cortisol was included as a covariate and in a third model additionally triglycerides. Moreover, we included the rs1360780 genotype as a covariate and tested the rs1360780 genotype and OC intake interaction term. For replication in SHIP-2, analogous models with the same specification were run. Finally, a combined regression analysis comprising both cohorts was performed, once again using the same covariates as before.

All p-values reported were derived from two-sided test. Statistical Analyses were performed in STATA 14/MP (STATA Corp., College Station, Texas).

### Data Availability

All data used in the manuscript can be requested via the online data application form of the SHIP study (https://www.fvcm.med.uni-greifswald.de/dd_service/data_use_intro.php). This is free of charge in general. The SHIP-TREND whole-blood transcriptome dataset is available at GEO (Gene Expression Omnibus) public repository under GSE36382.

## Electronic supplementary material

Supplementary Tables and Figures
