# Supplementary material for: Evidence for Stress-like Alterations in the HPA-Axis in Women Taking Oral Contraceptives
Source: Sci Rep. 2017 Oct 26;7:14111. doi: 10.1038/s41598-017-13927-7 (PMC5658328; doi:10.1038/s41598-017-13927-7)
Supplement: Supplementary file 1 — Supplementary Tables and Figures [file 41598_2017_13927_MOESM1_ESM.pdf]

Supplement for the manuscript

Evidence for Stress-like Alterations in the HPA-Axis in Women Taking Oral Contraceptives

Johannes Hertel[1,2*#], Johanna König[1*], Georg Homuth, Ph.D.[6], Sandra Van der Auwera[1,2], Katharina Wittfeld[2], Maik Pietzner[3,4], Tim Kacprowski[4,6], Liliane Pfeiffer[8,9], Anja Kretschmer Ph.D.[8,9], Melanie Waldenberger, Ph.D.[8,9], Gabi Kastenmüller[10], Anna Artati[11], Karsten Suhre[10,12], Jerzy Adamski[11,13,14], Sönke Langner[15],Uwe Völker, Ph.D.[4,6], Henry Völzke, M.D.[4,5], Matthias Nauck, M.D.[3,4], Nele Friedrich, Ph.D.[3,4,7#], Hans Joergen Grabe, M.D.[1,2#]

*contributed equally

[1] Department of Psychiatry and Psychotherapy, University Medicine Greifswald, Germany

[2] German Center for Neurodegenerative Diseases (DZNE), Site Rostock/ Greifswald, Germany

[3] Institute of Clinical Chemistry and Laboratory Medicine, University Medicine Greifswald, Germany

[4] DZHK (German Center for Cardiovascular Research), partner site Greifswald, Germany

[5] Institute for Community Medicine, University Medicine Greifswald, Greifswald, Germany

[6] Interfaculty Institute for Genetics and Functional Genomics, University Medicine Greifswald, Germany

[7] Research Centre for Prevention and Health, Glostrup University Hospital, Denmark

[8] Research Unit of Molecular Epidemiology, Helmholtz Zentrum München, German Research Center for Environmental Health, Neuherberg, Germany

[9]Institute of Epidemiology II, Helmholtz Zentrum München, German Research Center for Environmental Health, Neuherberg, Germany

[10] Institute of Bioinformatics and Systems Biology, Helmholtz Zentrum München, Neuherberg, 85764, Germany

[11] IEG (Institute of Experimental Genetics), Genome Analysis Center, Helmholtz Zentrum München, Neuherberg, 85764, Germany

[12] Weill Cornell Medical College in Qatar, Education City, Qatar Foundation, Doha, Qatar

[13] DZD (German Center for Diabetes Research), site München-Neuherberg, Neuherberg, 85764, Germany

[14] Lehrstuhl für Experimentelle Genetik, Technische Universität München, Freising-Weihenstephan, 85354, Germany

[15]Institute of Diagnostic Radiology, University Medicine Greifswald, Germany

Address for correspondence:
Johannes Hertel
Department of Psychiatry, University Medicine Greifswald, Germany
Ellernholzstrasse 1-2, 17475 Greifswald
Tel.: ++49 – (0)3834/ 86 22 1 66; Fax.:++49 – (0)3834/86 68 89
e-mail: hertelj@uni-greifswald.de

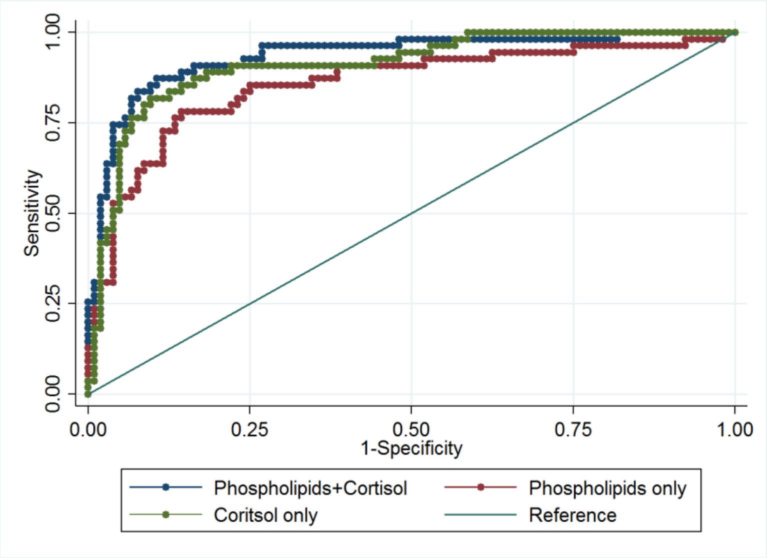

Figure S1: ROC curves for phospholipid classification of OC usage (red, area under the curve (AUC)=0.86), cortisol (green, AUC=0.91) and cortisol combined with phospholipids (blue, AUC=0.94). ROC curves are statistically different (p<0.01).

Table S1: Eigenvalues of the principle components of phospholipids

Principle Component	Eigenvalue
1	2.58
2	2.14
3	0.90
4	0.70
5	0.61
6	0.54
7	0.40
8	0.13

Table S2: Loadings of phospholipids on the first two principle components

	Principle Component 1	Principle Component 2
1-linoleoylglycerophosphocholine	0.49	0.36
1-palmitoylglycerophosphate	-0.33	0.26
2-linoleoylglycerophosphocholine	0.45	0.31
1-palmitoylglycerophosphoinositol	-0.31	0.36
1-palmitoylglycerophosphoethanolamine	-0.23	0.48
1-oleoylglycerophosphoinositol	-0.30	0.27
1-docosapentaenoylglycerophosphocholine	-0.43	0.30
1-docosahexaenoylglycerophosphoethanolamine	-0.15	0.43

Table S3: Association of phospholipid species and total triglycerides with OC usage. Sensitivity analyses

Compound	Mis., %	Model 1[a] b(95%-CI) for OC usage	p	N	Model 2[b] b(95%-CI) for OC usage	p
Cortisol	0.0	0.89 (0.73;1.06)	6.366e-21	197	0.95 (0.77,1.14)	4.881e-20
Triglycerides	0.0	0.37 (0.19; 0.56)	0.0001	200	0.41 (0.20;0.62)	0.0002
1-linoleoylglycerophosphocholine (18:2n6)	0.0	-0.24 (-0.38;-0.10)	0.0007	197	-0.24 (-0.39;-0.09)	0.0019
1-palmitoylglycerophosphate	8.3	0.35 (0.15;0.55)	0.0006	181	0.36 (0.14;0.58)	0.0017
2-linoleoylglycerophosphocholine	0.0	-0.24 (-0.39;-0.09)	0.0018	197	-0.26 (-0.42;-0.09)	0.0028
1-palmitoylglycerophosphoinositol	17.0	0.45 (0.30;0.59)	4.18e-09	161	0.43 (0.26;0.59)	6.215e-07
1-palmitoylglycerophosphoethanolamine	0.0	0.32 (0.19;0.45)	1.81e-06	197	0.37 (0.23;0.50)	1.578e-07
2-palmitoylglycerophosphoethanolamine	20.9	0.30 (0.14;0.47)	0.0004	156	0.34 (0.16;0.51)	0.0002
1-oleoylglycerophosphoinositol	9.6	0.35 (0.16;0.54)	0.0003	178	0.30 (0.10;0.51)	0.0044
1-docosapentaenoylglycerophosphocholine (22:5n3)	1.3	-0.42 (-0.61;-0.23)	2.58e-05	195	-0.43 (-0.64;-0.22)	8.40e-05
1-docosahexaenoylglycerophosphoethanolamine	0.0	0.22 (0.11;0.34)	0.0002	197	0.23 (0.11;0.35)	0.0003

[a]Model 1: adjusted for age, waist circumference, blood cell counts, fasting time, time of blood sampling and BDI-II-score
[b]Model 2: adjusted for age, waist circumference, blood cell counts, fasting time, time of blood sampling and BDI-II-score, MDD lifetime and CTQ score

Table S4: Top-Hits of the Transcriptome Wide Association Study (TWAS) on Circulating Cortisol Levels

Gene	beta	Standard Error	t	p[a]	Mean Intensity	Detection Rate	FDR[b]
DDIT4	0.30999	0.01978457	15.66828	0	8.525642395	1	0
FKBP5	0.19543	0.021948732	8.903933	2.98E-18	9.026628494	1	7.27E-14
CEBPD	0.121855	0.014438373	8.439684	1.28E-16	11.52982616	1	2.08E-12
TSC22D3	0.148345	0.019576032	7.577876	8.81E-14	10.81162262	1	1.06E-09
ECHDC3	0.141087	0.018700032	7.544766	1.12E-13	7.401368141	0.996742666	1.06E-09
DUSP1	0.178655	0.023745332	7.523806	1.3E-13	11.75978661	0.998914242	1.06E-09
IRS2	0.153778	0.021141438	7.27376	7.67E-13	8.539684296	1	5.34E-09
KLF9	0.132867	0.019235406	6.907442	9.39E-12	8.529838562	1	5.73E-08
FBXO32	0.080941	0.01340034	6.040214	2.26E-09	7.693478584	1	1E-05
CCND3	0.079452	0.013562377	5.858269	6.58E-09	12.50660133	1	2.6E-05
ZBTB16	0.120713	0.02063573	5.849691	6.91E-09	7.488571644	0.997828424	2.6E-05
TSC22D3	0.108573	0.018637262	5.82561	7.95E-09	7.473699093	0.992399573	2.63E-05
IL18R1	0.115021	0.019755004	5.822386	8.1E-09	8.12012291	1	2.63E-05
ZBTB16	0.098722	0.01703673	5.794641	9.5E-09	6.809102058	0.85233444	2.9E-05
CXCR4	0.097555	0.017459987	5.587322	3.06E-08	8.861386299	1	8.8E-05
RNF144B	0.088643	0.016143128	5.49104	5.21E-08	8.186312675	1	0.000141
ID3	-0.08677	0.016014749	-5.41837	7.75E-08	7.780823708	0.998914242	0.000199
SOCS1	0.075597	0.014539026	5.199621	2.48E-07	6.695757389	0.845819771	0.000576
IL7R	0.092921	0.018605242	4.994351	7.1E-07	10.64928913	1	0.001576
IL1B	-0.09457	0.019148158	-4.93899	9.38E-07	9.05814743	1	0.00199
CDKN1B	-0.06413	0.013474356	-4.75958	2.26E-06	9.012987137	1	0.004602
TLR10	-0.06783	0.014507816	-4.67511	3.39E-06	7.260742664	0.992399573	0.006368
IL1R2	0.096261	0.021317955	4.515485	7.17E-06	8.580068588	0.997828424	0.012491
HCAR2	-0.07182	0.016090393	-4.4634	9.1E-06	6.866519928	0.887079239	0.015317
GPR18	-0.06704	0.0151117	-4.43601	1.03E-05	7.56951189	0.996742666	0.016233
P2RY13	-0.06719	0.015176388	-4.42699	1.07E-05	10.27272224	1	0.016313
TSC22D3	0.09939	0.022480585	4.421144	1.1E-05	10.58773518	1	0.016313
TLR10	-0.07021	0.016108844	-4.35868	1.46E-05	7.028824329	0.952225864	0.020798
CDK5R1	0.084563	0.019539991	4.327712	1.68E-05	8.054044724	0.998914242	0.02274

BID	-0.05978	0.013950498	-4.28547	2.02E-05	8.100402832	1	0.026674
PASK	-0.08577	0.020225292	-4.24088	2.46E-05	7.918303967	0.997828424	0.030774
TLR2	0.061194	0.01460359	4.190306	3.06E-05	6.981621742	0.937024951	0.037378
HVCN1	-0.06801	0.016264427	-4.18122	3.19E-05	7.28487587	0.976112902	0.037925
SLA	0.067773	0.016288623	4.160729	3.48E-05	7.309864521	0.986970663	0.040323
ETS1	0.068334	0.016442554	4.1559	3.55E-05	11.27615261	1	0.040323
NFKBIA	0.069396	0.016787166	4.133865	3.9E-05	10.59403896	1	0.043305
SESN1	0.071551	0.017355151	4.122779	4.09E-05	7.553817749	0.997828424	0.044393

[a]uncorrected p-value
[b]FDR=false discovery rate

Table S5: Associations of *FKBP5* and *DDIT4* whole-blood transcript levels with OC usage. Sensitivity analyses.

Transcript	Mis., %	Model 1[a] b(95%-CI)	p	N	Model 2[b] b(95%-CI)	P
		**Main Effect (OC usage)**			**Main Effect (OC usage)**	
FKBP5	3.0%	0.10 (-0.01;0.20)	0.0660	195	0.12 (0.00;0.23)	0.049
DDIT4	3.0%	0.18 (0.08;0.28)	0.0004	195	0.19 (0.09;0.28)	0.0003
		**Interaction term (rs1360780xOC usage)**			**Interaction term (rs1360780xOC usage)**	
FKBP5	3.0%	-0.34 (-0.59;-0.09)	0.008	195	-0.31 (-0.60;-0.02)	0.038
DDIT4	3.0%	-0.01 (-0.25;0.23)	0.966	195	-0.01 (-0.27;0.26)	0.961

[a]Model 1: adjusted for age, waist circumference, blood cell counts, fasting time, time of blood sampling and BDI-II-score
[b]Model 2: adjusted for age, waist circumference, blood cell counts, fasting time, time of blood sampling and BDI-II-score, MDD lifetime and CTQ score

Table S6: Interaction effects of the rs1360780 genotype (CC/CT vs TT) and OC intake on DNA Methylation Levels in *FKBP5* Intron 7. Sensitivity analyses.

		Interaction term (rs1360780xOC usage)	
**Model**	**N**	**OR(95%-CI)**	**p**
[a]Model 1	229	1.22 (0.98;1.51)	0.069
[b]Model 2	233	1.24 (1.00;1.53)	0.048
[c]Model 3	200	1.20 (0.95;1.53)	0.130

[a]Model 1: adjusted for age, waist circumference, blood cell counts, fasting time, time of blood sampling and cortisol
[b]Model 2: adjusted for age, waist circumference, blood cell counts, fasting time, time of blood sampling and BDI-II-score
[c]Model 3: adjusted for age, waist circumference, blood cell counts, fasting time, time of blood sampling and BDI-II-score, MDD lifetime and CTQ score; note that Model 3 is **misspecified** as the CTQ score interacts itself with OC intake, Model 3 is only given for completeness.

Table S7: Descriptive Statistics for 303 pre-menopausal women of SHIP-2

	no OC usage	OC usage	p-Value
N	229	74	
Age (years)	42.8 (5.9)	40.8 (5.6)	0.007[c]
Waist Circumference (cm)	81.4 (11.9)	76.3 (7.5)	<0.001[c]
Triglycerides (mmol/l)	1.3 (0.9)	1.3 (0.6)	0.409[c]
rs1360780 (TT carrier, %)	7.86	10.81	0.474[d]
CTQ[a]	33.2 (12.8)	31.2 (8.0)	0.098[c]
BDI-II[b]	6.0 (6.5)	4.2 (5.9)	0.031[c]
Lifetime depression (%)	24.12	16.22	0.198[d]

[a]CTQ = Childhood Trauma Questionnaire
[b]BDI-II = Beck Depression Inventory II
[c]p-values from Welch t-test
[d]p-values from Fisher's exact test

Table S8: Interaction Effect between Childhood Trauma Questionnaire Scores and Intake of OCs. Sensitivity Analyses.

Model	SHIP-Trend-0 Interaction term (CTQxOC usage) OR(95%-CI)	p	SHIP-2 Interaction term (CTQxOC usage) OR(95%-CI)	p	Combined Interaction term (CTQxOC usage) OR(95%-CI)	p
Model 1[a]	1.01 (1.00;1.02)	0.002	1.01 (0.99; 1.01)	0.117	1.01 (1.00; 1.01)	0.003
Model 2[b]	1.01 (1.00;1.02)	0.003	1.01 (1.00; 1.02)	0.030	1.01 (1.00;1.01)	0.001

CTQ=Childhood trauma questionnaire; combined modelling included cohort as covariate
Model 1[a]**:** adjusted for age, waist circumference, blood cell counts, fasting time, time of blood sampling
Model 2[b]: adjusted for age, waist circumference, blood cell counts, fasting time, time of blood sampling, MDD lifetime and current depressive symptoms

Table S9: Associations of the hippocampal volume with OC intake. Sensitivity Analyses.

Model	SHIP-Trend-0 b(95%-CI) for OC usage	p	SHIP-2 b(95%-CI) for OC usage	p	combined b(95%-CI) for OC usage	p
Model 1[a]	-75.61 (-157.58; 6.36)	0.070	-42.41 (-142.11; 57.29)	0.402	-61.52 (-124.45; 1.41)	0.055
Model 2[b]	-84.07 (-166.57; -1.56)	0.046	-44.36 (-142.93; 54.20)	0.375	-67.36 (-130.07; -4.64)	0.035
Model 3[c]	-99.31 (-182.36; -16.26)	0.019	-48.00 (-148.21; 52.22)	0.345	-78.15 (-141.43; -14.88)	0.016
Model 4[d]	-179.47 (-285.74; -73.19)	0.001	-----	-----	-----	-----
Model 5[e]	-71.82 (-155.45; 11.82)	0.092	-44.15 (-143.57; 55.27)	0.381	-63.24 (-125.78; -0.70)	0.048
Model 6[f]	-98.19 (-180.80; -15.59)	0.020	-41.71 (-142.54; 59.12)	0.415	-78.10 (-141.56; -14.63)	0.016
Model 7[g]	-99.79 (-183.37; -16.21)	0.020	-46.39 (-146.64; 53.85)	0.362	-78.14 (-141.33; -14.96)	0.016
	**Interaction (SNPxOC)**		**Interaction (SNPxOC)**		**Interaction (SNPxOC)**	
Model 8[h]	-69.22 (-298.09; 159.65)	0.551	104.05 (-170.59; 378.70)	0.455	17.08 (-174.79; 208.95)	0.861

[a]Model 1: adjusted for age, intracranial volume, waist circumference and education
[b]Model 2: adjusted for age, intracranial volume, waist circumference, education and CTQ scores
[c]Model 3: adjusted for age, intracranial volume, waist circumference, education, CTQ scores and BDI-II scores
[d]Model 4: adjusted for age, intracranial volume, waist circumference, education, CTQ scores, BDI-II scores and circulating cortisol
[e]Model 5: adjusted for age, intracranial volume, waist circumference, education, CTQ scores, BDI-II scores and triglycerides
[f]Model 6: adjusted for age, intracranial volume, waist circumference, education, CTQ scores, BDI-II scores and SNP rs1360780
[g]Model 8: adjusted for age, intracranial volume, waist circumference, education, CTQ scores and BDI-II scores and MDD lifetime
[h]Model 7: interaction effect of SNP and OC intake, adjusted for adjusted for age, intracranial volume, waist circumference, education, CTQ scores, BDI-II scores

Table S10: Genotype distribution and Hardy-Weinberg Equilibrium for rs1360780 for subjects with MRI. The corresponding total genotype-sample (genotype distribution for SHIP-2 and SHIP-TREND, respectively) is given in brackets.

	CC	CT	TT	HWE (p-value)
SHIP-2	310 (1988)	278 (1742)	41 (334)	0.05 (0.08)
SHIP-TREND (Illumine HumanOmni)	326 (467)	296 (422)	64 (92)	0.86 (0.88)

Table S11. Sequences of PCR tagged primers used for EpiTYPER methylation analysis

Amplicon	Primer	Sequence	Product size (bp)	Total No. of CpG sites in amplicon
FKBP5	forward	aggaagagagTTGTTTTTGGAATTTAAGGTAATTGA	341	5
	revers	cagtaatacgactcactatagggagaaggctAAATCCAAAACTTATTCCCTTATTTA		
